# Validation of the integration of HIV and AIDS related nursing competencies into the undergraduate nursing curriculum in South Africa

**DOI:** 10.4102/curationis.v38i2.1521

**Published:** 2015-12-17

**Authors:** Regis R. Marie Modeste, Oluyinka Adejumo

**Affiliations:** 1 School of Nursing, University of the Western Cape, South Africa

## Abstract

**Background:**

Being in its fourth decade, HIV remains an epidemic that requires combined efforts for the global fight. The strategies planned and implemented in the fight against HIV include reversing and halting the spread of HIV, increasing health care access, and strengthening the health care system. South Africa has made the fight one of its top priorities, and has developed plans to increase the role of nurses in the management of HIV, demonstrating its willingness, commitment and progress in the fight against HIV.

**Objective:**

This article presents the validation process conducted to confirm the integration and mapping of the HIV and AIDS related nursing competencies into the four-year Bachelor of Nursing programme at a university in South Africa.

**Methods:**

This study adopted a constructivist paradigm, using a qualitative approach, applying the design step of the process model of curriculum development, to validate the integration of the mapped HIV and AIDS related nursing competencies into the undergraduate nursing curriculum.

**Results:**

For each competency, outcomes were developed for each year. Participants confirmed completeness of outcomes and appropriateness of the mapping of the HIV and AIDS related outcomes into the nursing curriculum, as well as the feasibility and practicability of the integration.

**Conclusion:**

Required resources for integration of HIV and AIDS related nursing competencies, such as human resources and nurse educators’ continued personal development were identified, as well as barriers to integration, and measures to eliminate them were discussed. The importance of integration of HIV and AIDS nursing competencies into the curriculum was reiterated.

## Introduction

With the human immunodeficiency virus (HIV) epidemic in its fourth decade, various countries and organisations have joined their efforts to fight the epidemic, by collaboratively designing and implementing a number of strategies. Increasing health care access, halting the spread of HIV, reversing it and strengthening the health care system are some of the strategies planned and implemented in the fight against the HIV epidemic (United Nations Programme on HIV and AIDS [UNAIDS] 2002:11; 2015:32). The World Health Organisation (WHO) has developed and supported a number of guidelines and policies, such as Integrated Management of Adolescent and Adult Illness (IMAI), Integrated Management of Childhood Illness (IMCI), prevention of mother-to-child transmission of HIV (PMTCT), and antiretroviral therapy (ART) guidelines (World Health Organisation [WHO] 2004; 2014). Furthermore, such policies and guidelines recommend task shifting that requires nurses to be trained for providing primary care for HIV (WHO 2014:50).

The South African government has demonstrated its willingness, commitment, and progress in the fight against the HIV epidemic by making this fight one of its top priorities (Department of Health [DoH] 2010:12). Some of the drives implemented include increasing the number of people tested for HIV, the introduction of ART fixed-dose combinations providing a less challenging regiment of medication, and the increased number of people started and maintained on treatment (WHO 2013:77).

In South Africa, similar to many other countries, nurses constitute the majority of health care workers (Dohrn, Nzama & Murrman 2009:S27; Flodgren *et al.* 2012:1). This makes the vital role of nurses in the fight against the HIV epidemic unquestionable, as evidenced by the various targets set to ensure the success of the various polices, guidelines, and goals that have been set. The 2007 to 2011 and 2012 to 2016 National Strategic Plans on HIV, sexually transmitted infections, and tuberculosis (NSPs on HIV, STIs, and TB) emphasise the goal of expanding the role of nurses in the management of HIV. The targets are related to the percentages of people to be started on ART, monitored, and maintained on treatment by nurses, striving to ensure earliest enrolment, as well as treatment for those people living with HIV (DoH 2007:86; South African National AIDS Council [SANAC] 2011:48). Nurses’ role is not limited to the provision of treatment and management; nurses participate in the prevention strategies too (Zulu & Lehmann 2004:21). However, a number of authors have noted that there are some limitations related to the preparation of nurses in terms of the care and management of patients living with HIV (Dohrn *et al.* 2009:S28; Knebel *et al.* 2008; McNabb *et al.* 2009; Zuber *et al.* 2014:521). Recommendations have been made previously by a number of authors to integrate HIV in the undergraduate Bachelor of Nursing programmes to facilitate successful care and management of people living with HIV and AIDS (Bharat & Mahendra 2007:108; Johns Hopkins Program for International Education in Gynecology and Obstetrics [JHPIEGO] 2009; Puplampu *et al.* 2014:260; Renggli *et al.* 2008:341; Seung *et al.* 2008:1208). Integration in the curriculum relates to knowledge, and skills are combined across discipline and subjects area, and the merger may vary in depth, type and purpose, supporting theoretical, applied and practical integration (Kachra and Schnietz 2007:478; MacMath, Wallace & Chi 2009:452).

In an article previously published by the authors of the current article, HIV and AIDS related competencies for nurse graduates in South Africa were presented; namely knowledge, policy, ethics, interdisciplinary approach, personal and professional development, holistic safe practice, and health education (Marie Modeste & Adejumo 2014a). However, the process of validating the HIV and AIDS competencies for nurses was not described as part of the previous publication. As noted by Fawcett *et al.* (1994), consumers and implementers should validate the intervention and provide feedback on the developed intervention. This article presents the validation carried out by involving the various categories of stakeholders and other interested parties to provide their input and feedback.

### Aim

This article presents the validation process conducted to confirm the integration and mapping of HIV and AIDS related nursing competencies into the four-year Bachelor of Nursing programme at a South African university.

## Research objective

The objective of the study presented in this paper was to validate the developed model for integration of HIV and AIDS related nursing competencies that will be embedded into the four-year Bachelor of Nursing programme at a South African university.

### Literature review

In an effort to avoid compartmentalised education that is not relevant to real-life situations, educators have proposed a curriculum that presents a unified holistic view of life, hence the introduction of curriculum integration (Haslegrave 2006:441; Relan & Kimpston 1991:4). Integration has a more inclusive interpretation in curriculum practice (MacMath *et al.* 2009:453; Relan & Kimpston 1991:5). Some authors (Bereiter 1984:77; Lachman & Pawlina 2006:457) refer to integration across domain skills: such as critical thinking, problem solving, reasoning, and reflective practice. Other authors refer to integration in relation to teaching and learning strategies, as well as the addition of topics and subjects in the curriculum without being classified as a separate discipline, such as nutrition (Touger-Decker 2004:198) and HIV (Stepleman *et al.* 2008:36). Integration of HIV and AIDS nursing competencies in a nursing curriculum provides the opportunity to maintain continuity of HIV and AIDS related content across the nursing undergraduate programme, allowing nursing students to start interacting with patients who are living with HIV and AIDS from the first year of the programme. This should happen when nursing students start developing lower level competencies and basic skills, such as communication skills and history taking, as noted by Haslegrave (2006:441) and as mapped in this study.

**FIGURE 1:**
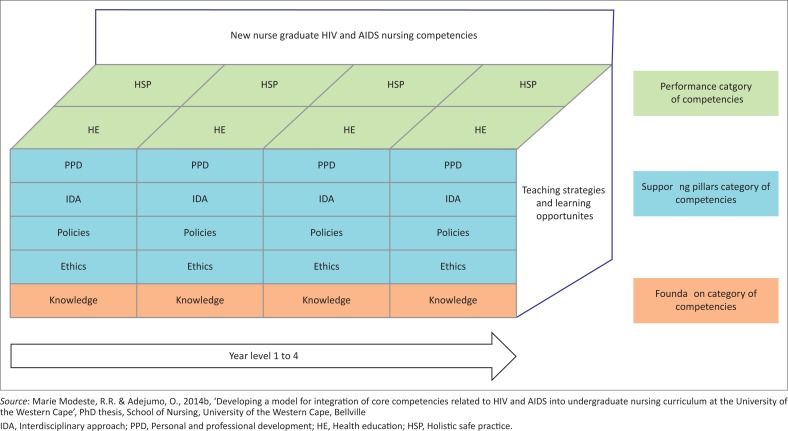
Vertical and horizontal integration of HIV and AIDS nursing competencies into a four-year nursing curriculum.

The first phase of the study developed the HIV and AIDS related nursing competencies for new nurse graduates, and the second phase of the study developed outcomes for each developed HIV and AIDS nursing competency for the four year Bachelor of Nursing programme, as well as the outcomes for each year level, providing vertical and horizontal integration. The vertical and horizontal integration of the HIV and AIDS nursing competencies as developed in this study (Figure 1) shows that each competency is carried forward from year one to year four, whilst simultaneously each competency is included in all the year levels of the nursing undergraduate programme.

Some criteria for successful integration have been documented (Ackerman 1989; Alberta Education 2007; Hagan, Ho & Hudis 2010). These criteria clarify whether it makes intellectual and practical sense to integrate specific aspects in the curriculum. The decision to integrate needs to be supported by intellectual criteria, such as the content integrity, validity of the aspects to be integrated within, for and beyond the discipline. The practical criteria include common planning time, budget, and flexible schedule for the integration, as well as support and personal commitment (Ackerman 1989; Alberta Education 2007; Hagan *et al.* 2010).

## Research design and methods

This study adopted a constructivist paradigm, which postulates that people develop subjective meanings of their experiences of the world in which they live. As these meanings differ, the researchers searched for complexity in meaning and multiple truths that existed within a context and were constructed by and between people (Bergman *et al.* 2012; Creswell 2014:8). De Villiers (2001:37) presented a three-phase process model of curriculum development that was applied in the study. The phases include planning, design and application, and this article is presenting the work completed in the design phase, implementing the process of deliberation and validation (De Villiers 2001:44). The process was implemented as a one-day workshop, followed by two electronic feedback opportunities. This article presents the validation process conducted as a third phase of a study that developed integration of HIV and AIDS nursing competencies into the Bachelor of Nursing curriculum. The first phase of the bigger study had developed the HIV and AIDS nursing competencies and related outcomes, whilst the second phase of the bigger study worked on the mapping of the developed competencies and outcomes.

### Population and sample

The population for this validation phase of the study included nurse educators from various universities that offer nursing undergraduate programmes and were involved in the development of the HIV and AIDS nursing competencies and mapping of the related outcomes, namely, nurses in the clinical setting in the Western Cape, recent graduates, people living with HIV as representatives from an organisation that provided care and support to people living with HIV and AIDS, as well as nurses with expertise in the field of HIV and AIDS. In addition, one registered nurse involved with the training of nurses during clinical practice and two people from the community who were not part of the development of competencies, were invited to participate. That was decided on to expose the work to people who had expertise in HIV and AIDS, with the purpose of adding a fresh perspective with the potential to increase the quality of the work. A nurse educator who is also a manager with expertise in HIV and AIDS was identified purposively; however, as a result of various work commitments, she was invited to provide feedback electronically.

Using volunteer and purposive sampling to recruit the participants, a total of 15 participants contributed to the validation process of the developed mapping of the HIV and AIDS related nursing competencies. Inclusion criteria included being a recent graduate of the institution for the recent graduate participants. For nurse educators, the criteria included working at a nursing school at university, and having participated in the development of HIV and AIDS related nursing competencies. For the expert nurse, the criteria were expertise and record of accomplishment in HIV work at a national and international level. For the registered nurses, the criteria were being involved in provision of care for HIV and AIDS, whilst the other was recruited based on her involvement in training for HIV and AIDS for in-service training of nurses. Characteristics of participants in the study are summarised in Table 1. There were 13 participants in the validation workshop, whilst two other invited participants who were unable to attend the workshop because of work commitments gave electronic feedback. Most participants (12) were from the Western Cape, with one participant each from KwaZulu-Natal, the Free State, and Gauteng provinces. Of the participants, 9 were aged 50 years and below, whilst the other 6 were older than 50. In total, 8 of the participants in the validation process had participated in the development of competencies.

**TABLE 1:** Characteristics of participants – *N* = 15.

Variable	Characteristic	Number of respondents
Category	Recent graduate	1
	Registered nurse	1
	Nurse educators	11
	Community member	1
	Expert nurse	1
Institution	University	11
	Clinical practice	2
	Community	1
	Nursing organisation	1
Qualification	PhD	3
	Masters’ degree	7
	Bachelor degree	2
	Basic 4 year Diploma	1
	Postgraduate Diploma (Nursing education)	1
Province	KwaZulu-Natal	1
	Free State	1
	Gauteng	1
	Western Cape	12
Contribution	Workshop	13
	Electronic feedback	2
Age group	50 years and less	9
	Over 50 years	6
Participated in previous phases of the study		9
Did not participate in previous phases of the study		6

### Data collection

Data were collected during a one-day workshop, facilitated by one of the authors. The workshop was conducted in English, as all the participants were conversant in that language. The participants were informed about the objectives of the workshop. After introducing the study and obtaining consent and completion of biographical details, a presentation was conducted about the study, highlighting the product of the first two phases of the study, namely the developed HIV and AIDS related nursing competencies with related outcomes, as well as how the outcomes were mapped into the nursing curriculum. Having applied the process model for curriculum development, discussions and deliberations took place. This fits the understanding that curriculum work is not linear but a process that requires regular checks to assess what ground had been covered previously as part of continuous evaluation (De Villiers 2001:44). The workshop provided the opportunity for discussions that were congruent with the constructivist philosophical approach applied in the study as a basis for the work on the curriculum, as well as in the research process (Denzin & Lincoln 2003; Wheelahan 2010).

### Workshop process

The workshop was conducted in two sessions, and in each session different questions and discussions were conducted as illustrated in Table 2. The first session of the workshop involved a review of the first document used in the workshop, which was a list of HIV and AIDS related nursing core competencies, sub-competencies, and related exit level outcomes for the four-year undergraduate Bachelor of Nursing programme. The participants were requested to check the document to establish completeness, accuracy and appropriateness. In addition, participants looked at the logical flow of outcomes specific for each competency, identified modifications that could be made on the competencies and related outcomes, as well as establishing if the competencies and related outcomes were communicated effectively. After feedback, discussions were conducted and agreement was reached on suggestions from the first session.

**TABLE 2:** Process followed in the workshop.

Session	Aim	Questions
1	Review of the HIV and AIDS core competencies for the program and related outcomes (Document 1).	Do the HIV and AIDS related competencies listed reflect all the required competencies for a new nurse graduate to be able to provide care and management for HIV and AIDS upon graduation? Could you identify competencies that should be added to or removed from the list? Are the HIV and AIDS related competency statements written to most effectively communicate performance expectations?
2	Review of specific competencies and outcomes for each year level (Document 2). Document 3 was used as an answer sheet. Establishing the feasibility and practicability of the developed integration.	Are the competency statements allocated to each year level appropriate? What is your view of the developed integration with regard to: Feasibility. Practicability. Resources. Barriers and measures to eliminate barriers.

The second session was used by participants to look at the outcomes that had been mapped for the four-year Bachelor of Nursing programme, using the second document used in the workshop. The second document provided specific HIV and AIDS related nursing competencies and outcomes for each year level of the programme. A third document was given to the participants as an answer sheet, and they were requested to establish the appropriateness of the competency statements developed for each year level as presented in the second document, by indicating if the statements are ‘not at all’, ‘somewhat’, ‘moderate’ or ‘completely’ appropriate. In the second session, the participants were divided into four groups. Again, the participants checked the documents for completeness, accuracy, logical flow, appropriateness for each year level, and whether the vertical integration over the four-year period was maintained for each competency. After discussion in small groups, the participants gave feedback and the whole group reached agreement by commenting on the feedback.

A further discussion was conducted about the feasibility and appropriateness of the developed integration model of nursing competencies related to HIV and AIDS into the four-year Bachelor of Nursing programme. In addition to the workshop participants, two participants who were invited but were unable to attend the workshop were asked to review the mapped competencies and to provide electronic feedback. They were provided the same documents and questions for consideration as those used during the workshop. Their comments were incorporated with the comments obtained during the workshop for the purpose of developing the final list of competencies and for mapping them into the curriculum.

### Data analysis

The researchers kept all written and electronic data safe and only the researchers had access to the data. Verbatim transcriptions were made of all the recorded data. Transcripts of recorded data were combined with the written data for complete analysis. Data were analysed using a qualitative content analysis method following the steps outlined by Terre Blanche, Durrheim and Painter (2006), and a deductive approach was applied based on the questions that were asked in the workshop. The main themes from the data were related to completeness of the HIV and AIDS related nursing competencies and the developed outcomes, adjustments to be made, and review of year level outcomes. Furthermore, themes related to feasibility and resources and barriers to integration were derived from the data.

## Results and discussion

### Participants’ expertise in nursing practice

The participants in this validation process brought their expertise in terms of HIV and AIDS, as well as nursing education. The participants’ expertise was related to their experience in practice where they were providing HIV and AIDS care and management to persons living with HIV and AIDS, including but not limited, to HIV counselling and testing, health education, screening and testing adults and babies, as well as working in rural areas.

### Participants’ expertise in HIV and AIDS related issues

In addition to nursing practice, another area of expertise was related to HIV and AIDS courses that the participants had attended. This included a master’s degree in HIV nursing, taking part in developing HIV and AIDS protocols and policies at local and national levels, managing an HIV and AIDS project in the Southern Africa region, in addition to participating in a nursing magazine that focuses on HIV in South Africa. Personal experience of living with HIV was also reported, as well as participation in research projects about HIV. Furthermore, participants reported experience in teaching HIV and AIDS to nurses at undergraduate and postgraduate level, as well as in-service training that included various aspects related to HIV and AIDS, such as prevention of mother-to-child transmission of HIV.

### Participants’ expertise in nursing education

The participants reported many years of practice as nurse educators, including teaching at diploma, undergraduate, and postgraduate levels, ranging from 2 to 27 years. This included 22 years of experience as a trainer of HIV and AIDS, TB, and STIs in-service training for nurses in clinical practice. The teaching experience related to HIV and AIDS was reported in various areas of nursing practice, such as midwifery and community health nursing. There were participants who also reported experience in teaching methods, such as case-based education and the skills-lab method, in addition to coordination responsibility in the Bachelor of Nursing programme.

### Completeness of the HIV and AIDS related nursing competencies and related outcomes

In the first session, participants were first asked to review the presented HIV and AIDS related core competencies and subsequent outcomes for the four-year Bachelor of Nursing programme. All participants responded positively and indicated that the nursing core competencies and related outcomes related to HIV and AIDS that had been developed met the requirements of what was expected of a new nurse graduate to competently provide care and management for HIV and AIDS patients, and adjustments were provided to increase clarity as outlined in the next session.

### Adjustments recommended on the broad HIV and AIDS related nursing competency statements

The second question asked the participants to identify competencies that could be added or removed. Although no additions or exclusions were reported, respondents indicated some modifications that should be made to the competencies, and each time they were asked to provide the reasoning behind their decisions. Three main adjustments were recommended, and these related to the knowledge, policy and holistic safe practice core competencies. The suggested modifications also included re-ordering the specific competencies into the knowledge and holistic safe practice core competencies related to assessment with the purpose of ensuring a logical flow as happens in practice. This is illustrated from the following statement:

‘… [*W*]e said assessment according to systematic approach involves obtaining history, looking at clinical features, and thirdly, coming to what is here as part of assessment. Assessment has got three aspects, isn’t it? You obtain history. Do physical examination to determine certain clinical features and then you confirm with the diagnostic tests. So we felt you should add obtaining history relating to HIV and AIDS and determining clinical features associated with HIV and AIDS.’ (P5)

#### Knowledge competency

The adjustments were to ensure comprehensiveness, clarity and logical presentation that fit practice. To ensure comprehensiveness, it was suggested that the explanation of the knowledge core competency should be expanded to include all aspects that were part of the competency. Furthermore, participants suggested removing ‘South Africa’, as knowledge is global and thus the issues covered in the knowledge core competencies would also be highly informed by aspects that are relevant globally, as explained in this excerpt:

‘And then it says something about relevant in South Africa, so I think you should take that out because it should be … it’s globally based. And then with the specific competencies, we rearranged them. We put them in order of importance. So the first one was to be scientific knowledge, then it should be health promotion and prevention, then we put assessment and then we grouped management with HIV-related diseases.’ (P2)

#### Policy competency

It was also suggested for the policies core competency that the explanation should be modified to ‘legislation and policies’ instead of ‘policies and regulation’ as stated by one participant:

‘Then the next one which is ethics, we were happy. And then with the policies, and we added legislation and scrapped regulation.’ (P9)

This was agreed to as it was understood that legislation guided policies and not the other way around.

Another suggestion was to include protocol as part of the second specific competency under policies. This is illustrated from the discussion from one of the participants:

‘And then with the second one we thought that you need to add in the word “protocol” but it must first be policies, because that is national events and then protocol because that is institutional events.’ (P4)

Furthermore, it was suggested to add leadership as part of the policy core competency, as indicated by this excerpt:

‘I would like to suggest that an element of leadership be added to the competencies, and this is specifically that new graduates should not only analyse and implement policies, but they should also analyse and influence policies. South African nurses do not sufficiently participate in opportunities to influence policies – as practitioners implementing policies; they are in an ideal position to influence policies.’ (electronic feedback 1)

#### Holistic safe practice competency

It was suggested and accepted that counselling should be combined with assessment. This change was substantiated from a practice point of view, where the advise, counsel, test and support (ACTS) framework for counselling and testing for HIV is currently used, as noted by one participant:

‘I want to say, it really has to do with the ACTS method that we are advice, counsel, and test and support. It is just plain and simple.’ (P5)

Nurses were encouraged to integrate the framework with their assessment of all patients in their care. The understanding was that separating counselling and assessment in the programme might create students’ impression that the two aspects were addressed separately. The aim was to ensure that counselling for testing was offered to all patients, in the hope of increasing the number of people who were tested for HIV.

#### Personal and professional development competency

During the workshop, there was also a debate about combining continual personal development and continual professional development. After discussion and voting on the issue, it was agreed to keep the two aspects separate as voted by 10 out of the 13 participants. The reasons for keeping the two aspects separate were that the personal aspects related to the nurses’ ability to prevent infection in their own lives and to manage the HIV infection appropriately, if infected. On the other hand, professional development aspects related to keeping themselves up to speed with the latest practice of required HIV and AIDS care and management were seen as being separate from personal development aspects. The two aspects are important, considering that the literature reports that about 15.7% of health care workers at public and private health care institutions in South Africa were living with HIV in 2004 (Shisana *et al.* 2004:849). Furthermore, 13.7% of nurses at two hospitals in South Africa were living with HIV in 2005 whilst 12% of females tested were HIV positive compared to 7.9% of males (Connelly *et al.* 2007:117). Additionally, Shisana *et al.* (2004:849) noted that health care workers in the 18 to 35 year age group had a higher HIV prevalence of 20% compared to the 13.7% of the 36 to 45 year age group, whilst the study by Connelly *et al.* (2007:118) reported that HIV prevalence for health care workers who were between 25 and 34 years was 15.9%, compared to 13.0% of the ones in the 35 to 44 year age group.

Also, as most of the nurses in South Africa are female (South African Nursing Council [SANC] 2014) and, like in many other sub-Saharan countries, females have an increased risk of infection (United Nations Programme on HIV and AIDS [UNAIDS] 2014), the personal development aspect is important to attend to during training. Connelly *et al.* (2007:117) also noted that 13.8% of nursing students in the two hospitals where they conducted the study were living with HIV. Hence, it is important to equip nurses whilst they were still in training about the avoidance of infection and managing themselves if infected with HIV. Furthermore, considering the fast pace of new information related to HIV and AIDS, the professional development is important to ensure that nurses remain well equipped to enhance their competency levels even after graduation, without relying solely on in-service training. However, one participant indicated that the core competency of personal and professional development was unsuitable for the undergraduate programme.

As the other 14 participants had included this core competency in the undergraduate Bachelor of Nursing programme, the core competency was maintained in the list of identified core competencies. Furthermore, nurse graduates need to be able to maintain their personal development and keep themselves healthy in terms of preventing HIV infection, managing themselves with regard to accessing and remaining in care if infected with HIV, as well as keeping up to speed with all the new information and changes in policies that are related to HIV and AIDS. For nurses to be competent in this area, the nursing education and training that they receive should enhance the development of the competency.

### Recommended additions to the competencies presentation format

The participants suggested that the presentation of the list of core competencies, specific competencies, and related outcomes should include the examples of content and context that would be addressed in each specific competency related to HIV and AIDS for nurses. This was accepted, as it would enhance the understanding of the details that would be considered in the teaching, learning, and development of those competencies. An example was given with regard to local policies; the participants expressed the need to indicate that there were institutional, provincial, and national policies and guidelines that were implemented in practice. In addition, with regard to assessment the participants in the workshop indicated the need to clarify that comprehensive assessment included history taking and physical assessment. The recommendations were integrated in the finalisation of the document, and further integrated by modifying the various outcomes of the year levels in terms of the HIV and AIDS competencies that had been developed in the study.

### Communicating the competency and outcomes effectively

Once the list of core competencies and specific competencies had been reviewed, the participants reviewed the outcomes developed for each specific competency and answered the third question: ‘Are the HIV and AIDS related competency statements written to most effectively communicate performance expectations?’ Participants agreed that the competency statements effectively communicated the performance expectations. The participants provided feedback such as adjusting the verb to fit the national qualification framework and Bloom’s taxonomy, and these were considered and integrated in the finalisation of the HIV and AIDS core competencies.

As part of the validation, the participants – who were divided in four groups – reviewed the mapping of the HIV and AIDS related nursing competencies into the four-year Bachelor of Nursing programme. This process produced four answer sheets, and an additional two answer sheets that were submitted by the two participants who provided electronic feedback. The fourth question of the review which dealt with appropriateness and completeness of the outcomes developed for each core competency in each year level, yielded six answer sheets. Of the six review answer sheets from which the developed core competencies, specific competencies, and outcomes were mapped into the four year levels, five indicated that it was carried out completely, as indicated by one participant:

‘In my view, the competencies have been suitably divided over the four-year programme.’ (electronic feedback 1)

Another participant stated:

‘You find also the things that they are appropriate to be done at that level.’ (P1)

The participants provided suggestions about the order and formulation that included a verb of some competencies to reflect the national qualification framework level by using Bloom’s taxonomy. Furthermore, suggestions were made about adjustments that would correspond with the ones that were made in questions two and three of the review.

One of the reviewers commented on year levels three and four based on the way the undergraduate Bachelor of Nursing programme was structured at the institution where the participant was based, and some of the suggestions were not implemented. An example of such a suggestion was to move some outcomes from the third to the second year because students did not have access to the community, as community health was part of the second year programme at that specific institution:

‘Please note: third year students in hospital setting – limited community exposure.’ (electronic feedback 2)

Suggestions like that one were not implemented, as the mapping was for a Bachelor of Nursing programme at a selected university where students had exposure to the community during their third year of training.

In terms of all the other comments, where the specific competency was ranked as ‘somewhat’ or ‘not at all’ important, suggestions such as adjusting the order in which competencies are presented were made and incorporated into the formulation of the outcomes for each year level. There were no other suggestions to move any of the outcomes from one year level to another, except for the one that was mentioned earlier and did not apply to the selected university Bachelor of Nursing programme. As the feedback received from the first round provided more than 80% agreement, the researchers did not send the list of core competencies, specific competencies, and outcomes for another round of feedback. Furthermore, as the suggestions made during the workshop were discussed and agreement was reached in the workshop, there was no need to resend the document to the participants.

### To integrate or not to integrate HIV and AIDS core competencies into the undergraduate nursing curriculum?

A number of authors have documented criteria for successful integration, stating that the aspect to be integrated into a curriculum must have the potential to contribute to broader outcomes of the programme, and that it must be feasible and possible within the financial and time limits of the programme. In addition, the commitment and support of the teaching staff are requirements for implementing the integration (Ackerman 1989; Hagan *et al.* 2010).

With that in mind, the researchers included questions to establish the feasibility, practicability, and support for the integration of HIV and AIDS into the undergraduate Bachelor of Nursing programme at a selected South African university.

### Feasibility and practicability

All participants indicated that it was feasible and practical to implement the developed integrated HIV and AIDS related nursing competencies into the undergraduate Bachelor of Nursing programme at the selected university. The participants commented that the developed HIV and AIDS competencies for nurses were appropriate and necessary, as nurses needed to be trained at all levels for the care and management of HIV and AIDS patients, as noted by one participant:

‘Great idea, students should be able to demonstrate their knowledge about the above.’ (P3)

Another noted:

‘All topics and information is relevant to be incorporated into the nursing curriculum.’ (P2)

Furthermore, participants indicated that the outcomes could be integrated into the case-based part of each course in the outcome based education approach applied at that school of nursing. This was possible because the outcomes were relevant to the undergraduate programme and they were viewed as being clear, increasing potential for integrating them into the curriculum.

The researchers took great care to avoid repetitions, as there is a need for integrating HIV and AIDS core competencies into the Bachelor of Nursing programme at undergraduate level. The HIV and AIDS epidemic remains a serious health problem in South Africa as illustrated by these excerpts from the validation phase of the workshop:

‘Definitely needed as epidemic is big and nurses at all levels need to be educated about HIV/AIDS.’ (P7)

And:

‘All levels are considered, competencies for each level leads to the next excluding repetitions.’ (P7)

For successful implementation of integrating HIV and AIDS nursing competencies into the undergraduate Bacheor of Nursing programme at a selected South African university, the respondents indicated that sufficient human resources should be available. That referred not only to enough teaching staff for theory and practice, but also for attending to the teaching staff’s development by means of courses, workshops, and in-service training; staff development had the potential to enhance the teaching staff members’ ability to perform to the highest standards. That was illustrated by the following excerpts from respondents during the validation phase:

‘More human power well versed with [*sic*] the integration of competencies in the programme, … in-service training.’ (P5)

Another indicated that competent teaching staff formed part of the resources that were needed for successful integration:

‘Competent and well informed lecturers.’ (P11)

Equipment was also mentioned as a requirement, such as the necessary equipment in the clinical settings for nursing students’ training and practice, as well as audio-visual training material and use of media as illustrated by one respondent:

‘Models of HIV, those that can be taken apart and reconstructed again.’ (P7)

And another who noted:

‘Efficient equipment in ward and clinical setting.’ (P9)

### Eliminating barriers to integrating of HIV and AIDS core competencies

During the third phase of the study, participants were asked to identify barriers to implementation and how those barriers could be eliminated. The responses indicated that staff development and provision of teaching staff would eliminate barriers related to lack of staff, as well as lack of knowledge of the teaching staff. Some respondents indicated:

‘Upgrade courses for lecturers once or twice for all levels.’ (P7)

‘… all lecturers to be trained in HIV/AIDS and its implication in society.’ (P13)

‘… workshops to be conducted at least once or twice a year for all year levels.’ (P7)

Furthermore, provision of learning opportunities to practice and sufficient resources would support the success of the integration.

### Academic rigour

Trustworthiness in this qualitative study was achieved by adherence to a number of criteria during data collection and analysis processes to ensure truth-value, applicability, consistency, and neutrality of the findings, as well as the credibility, transferability, dependability, and confirmability of the concepts that were assessed (Klopper & Knobloch 2010). Trustworthiness was obtained by means of prolonged engagement, as many of the participants in the validation process had participated in the study from the beginning. The inclusion of a variety of participants with the aim of obtaining a wide range of perspectives ensured confirmability as triangulation was applied. Furthermore, the crosschecking undertaken by the two authors allowed enquiry audit ensuring dependability and confirmability (Klopper & Knobloch 2010; Polit & Hungler 1997).

## Conclusion

The combination of experiences enabled participants to review the mapping that was carried out for the HIV and AIDS-related nursing core competencies and establish relevance and adherence to educational principles. The variety of expertise from the participants provided a rich source of information and an opportunity to view the study from various angles. All participants brought their own perspectives, and the combination of their views enhanced the outcome of the review conducted with the purpose of integrating HIV and AIDS related nursing competencies into the undergraduate Bachelor of Nursing programme.

The participants confirmed and supported the identified competencies related to HIV and AIDS for nurses, and they viewed them as being appropriate, complete, and relevant for the newly graduated nurse. The outcomes and the mapping of the outcomes in the four-year Bachelor of Nursing programme at a selected South African university were also reviewed; participants supported the presented integration provided that relevant adjustments were made and adopted. The adjustments were useful, as they provided opportunities to present the outcomes logically and incorporating them into the nursing students’ current practice situations.

The workshop participants also viewed the integration of HIV and AIDS core competencies into the undergraduate Bachelor of Nursing programme at a selected university as feasible and practical, as well as important for the training of nurses who provide care and management to patients in the South African context. Table 3 presents an extract of the core competencies that were validated, and one specific competency and its related outcome for each core competency. Furthermore, this article has presented a systematic approach of involving a variety of stakeholders in enhancing the integration of HIV and AIDS competencies, by reviewing, validating and verifying the prepared work. Integration of HIV and AIDS care and management has been noted as a weakness in many curricula implemented in the training of nurses before entering practice, despite the 2007 recommendation by the WHO (Dohrn *et al.* 2009:S28; McNabb *et al.* 2009; Renggli *et al.* 2008:341; Seung *et al.* 2008:1208; WHO 2002:11). This provides an opportunity to have a complete and appropriate mapping of related outcomes in the various year levels in the nursing undergraduate programme at one university in South Africa.

**TABLE 3:** A sample of HIV and AIDS nursing competency statement, specific competency, outcomes and related concepts.

Competency category	Competency area	Specific competency	Outcomes	Examples of related concepts/content
Foundation	Knowledge: Knowledge about care and management, scientific knowledge, health promotion and prevention, as well as issues related to HIV and AIDS.	Basic scientific knowledge about HIV and AIDS.	Evaluate the basic scientific facts about HIV and how it is applied in the care and management of HIV.	HIV-related terms and concepts, history, microbiology and pathophysiology, epidemiology, myths.
Supporting pillars	Ethics: Ethics related to HIV and AIDS in the care and management of patients living with HIV and AIDS for the reduction of stigma and increase in patients’ positive experience.	HIV and AIDS ethics-related issues	Correctly and appropriately deal with ethical dilemmas related to HIV and AIDS and adhere to and monitor the correct application of ethics on HIV-related research.	Ethical principles.Ethical dilemmas.
	Policies: Legislation and policies related to HIV and AIDS when caring for various types of patients living with HIV and AIDS in different settings.	Legislation	Describe and follow the legal requirements as regulated in the care of patients living with HIV and AIDS.	Legislation related to HIV and AIDS: Confidentiality and disclosure, eligibility criteria, Mental Health Care Act.
	Interdisciplinary approach: Interdisciplinary approach in the care and management of patients living with HIV and AIDS.	Community involvement.	Participate in community engagement, programmes and interventions within a collaborative framework enhancing involvement with NGOs, FBOs and CBOs in the care and management of HIV and AIDS.	Community engagement principles and approaches.
	Personal and professional development: Personal and professional plan for continuous development and care of the carer as a health care provider for clients affected by and infected with HIV and AIDS.	Continuous personal development.	Develop a continuous personal development plan and take responsibility to apply the learned information and skills in own life for the prevention and management of HIV.	Self-awareness, clarifying own beliefs and values, emotional readiness.
Performance	Health education: Health education and promotion related to HIV and AIDS to different groups of clients that are at risk of getting infected, infected with HIV and those affected by HIV and AIDS.	Information transfer.	Appropriately transfer information related to HIV and AIDS to others and facilitates learning, taking into consideration various relevant aspects such as culture and context.	Information transfer.Facilitation of learning.
	Holistic safe practice: Holistic and safe care and management for patients living with HIV and AIDS.	Interpersonal skills.	Demonstrate effective communication, interviewing and motivational skills in the care and management of HIV.	Communication and interview skills, observation skills, therapeutic communication.

### Recommendations

The study recommends that the nurse educators in various schools review the various outcomes for each year level, rearrange them to ensure fitness into their programmes, and then implement the integration based on the relevancy to their programme and health care needs of the communities they serve. Nurse educators should consider the learning opportunities available for the student nurses to develop the HIV and AIDS related nursing competencies, in order for them to effectively provide care and management related to HIV and AIDS upon graduation.

Possible barriers and solutions were presented, and as successful integration depends on buy-in from the educators involved in the teaching, the study recommends staff development sessions for the teaching staff on HIV and AIDS care and management, and on teaching and learning strategies, to support integration, maximise the integration, and facilitate enhanced development of HIV and AIDS competencies. As information on HIV evolves continuously, these staff development sessions will also provide opportunities to inform the nurse educators on changes that are relevant in the field of HIV and AIDS, in addition to evaluating the process regularly to fill any possible gaps.

## Ethical considerations

The study received ethical clearance and permission from the various institutions where the participants were recruited. All participants were given an information document, and signed consent forms were obtained. In the study, participants’ anonymity and confidentiality were maintained. All participants were informed of their right to withdraw at any time if they wished. Furthermore, permission for audio recording of the workshop was obtained.

### Limitations of the study

The limitations of the study include the inability to involve more people in the workshop owing to limited financial means. To counteract this limitation, electronic feedback was obtained from two experts in the area of HIV and AIDS, as well as nursing education, as they reported not being able to travel for the workshop. Furthermore, the validation was completed based on one Bachelor of Nursing programme in South Africa. As the curricula are not always structured in the same way, the integrated competencies cannot be adopted exactly as validated in this study. However, because outcomes apply to year level of training and nursing speciality areas are visible, adjustments can be customised for any other nursing undergraduate programme.
